# The therapeutic approaches of renal recovery after relief of the unilateral ureteral obstruction: A comprehensive review

**DOI:** 10.22038/ijbms.2020.41984.9926

**Published:** 2020-11

**Authors:** Ayat Kaeidi, Maryam Maleki, Ali Shamsizadeh, Iman Fatemi, Elham Hakimizadeh, Jalal Hassanshahi

**Affiliations:** 1 Physiology-Pharmacology Research Center, Research Institute of Basic Medical Sciences, Rafsanjan University of Medical Sciences, Rafsanjan, Iran; 2 Department of Physiology and Pharmacology, School of Medicine, Rafsanjan University of Medical Sciences, Rafsanjan, Iran; 3 Department of Physiology, Ilam University of Medical Sciences, Ilam, Iran; 4 Research Center of Tropical and Infectious Diseases, Kerman University of Medical Sciences, Kerman, Iran

**Keywords:** Animal, Human, Recovery, Relief, Therapeutic procedure, Unilateral ureteral, obstruction

## Abstract

Unilateral ureteral obstruction (UUO) as a clinical disorder can cause renal damage. The permanent injury occurs if the obstruction is not relieved. Renal injury can be reversed with UUO removal (RUUO). RUUO attenuates the renal hemodynamic and functional impairment and decreases the renal fibrosis and apoptosis. Nevertheless, kidney injury may continue after RUUO, and synchronous medication therapy seems necessary. However, UUO and post-RUUO periods are also important in final renal recovery. To date, various therapeutic strategies have been applied to develop renal recoverability after RUUO. In animal studies, the effect of some pharmacological agents such as mesenchymal stem cells, anti-inflammation drugs, L-arginine, bone morphogenetic protein-7, epidermal growth factor, allopurinol, renin-angiotensin system antagonists, and endothelin A/B receptor blocker were surveyed in RUUO model. Also, post-RUUO renal recoverability has been studied in human researches. In these studies, the effective strategies have focused on surgery for RUUO creation via urethrotomy, urethroplasty, stent balloon dilatation, and stenting. Accordingly, in this review, we focused on the therapeutic procedure of renal recovery after the RUUO situation in human and animal studies.

## Introduction

**Figure 1:**
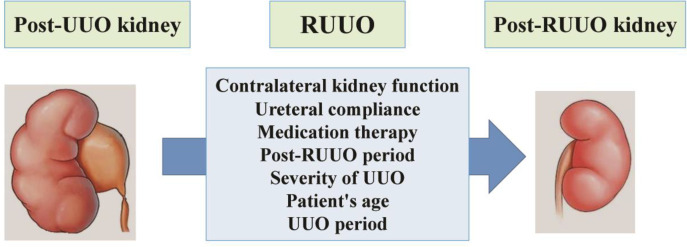
The main factors affecting on ipsilateral kidney recovery in animals and humans subjected to RUUO

Ureteral obstruction is known as one of the common clinical renal disorders that can occur at any age (1). It has been shown that if ureteral obstruction continues, it causes nephropathy (2) and chronic kidney injury (3) and if detected promptly can be treated and reversed (1). The ipsilateral ureteral pressure increases in the unilateral ureteral obstruction (UUO) model (4). UUO reduces blood supply to the ipsilateral kidney (2) and consequently, its glomerular filtration rate (GFR) is reduced (4, 5), the cellular and molecular abnormalities appear in the obstructive kidney (6, 7) and ultimately progresses to fibrosis (8). These conditions like other pathologic situations can induce kidney injury (9-11). The permanent injury occurs when obstruction is not relieved for a long time (12). Many UUO studies have shown that the safest way to prevent ipsilateral kidney injury is UUO removal (RUUO) as quickly as possible (13, 14). The renal response to RUUO depends on several factors including the duration and severity of UUO, patient’s age, ureteral compliance, and the contralateral kidney function during UUO (Figure 1) (4). Many animal and human studies have been performed on renal hemodynamics (4, 15), function, and injury after RUUO (13, 14). To date, the cellular and molecular mechanisms of UUO are being investigated, while renal intrinsic mechanisms have been less evaluated following RUUO (16). However, UUO with long duration causes fibrosis and apoptosis in ipsilateral kidney, and it is less likely to be completely reversible (17), whereas UUO with short duration (<24 hr) can be completely reversible (17). Also, after the removal of short-term UUO, the number of aquaporin channels (18), sodium transporters (19), and GFR returned to normal within two weeks (16). Moreover, it has been observed that the number of ipsilateral kidney macrophages is reduced within 4 weeks after RUUO in the animal with a short-term UUO model (16). Since some reports suggested that kidney injury can continue even after RUUO (20-22), probably RUUO alone is not enough to recover the renal function (17, 23) and its histopathology, and concurrent drug interventions seem necessary (24). Furthermore, it has been determined that free radicals can be produced even after RUUO and contribute to renal ischemia via the reduction of renal blood flow (RBF) (25). Generally, RUUO and accompanying interventions can change the complex equations of renal injury to repair and cause a balance between cell loss and proliferation (12, 16). Today, various studies have suggested different treatments to improve kidney function after RUUO (15, 26). Accordingly, this review focuses on the therapeutic approaches to renal recovery after RUUO in animal models and human researches.

***The necessity of RUUO creation and medication therapy***

UUO causes urinary sediment, debris, and retention in renal tubules (5, 27), so that after nephrogenesis, the ipsilateral kidney is susceptible to permanent injury (28). Today, it is known that the crucial treatment of UUO is relief of obstruction (29), and RUUO is as important as detecting obstruction (28). However, sometimes the determination of obstruction is a problem for clinicians (29). After RUUO, the compression is removed from the ipsilateral kidney (27), and with the starting and continuing of drainage, the kidney decompression is completed (Figure 1) (27). In this regard, it has been shown that the increase in renal function recovery is an important issue after RUUO (17). Also, it has been reported that the ipsilateral renal injury can continue even after RUUO (17). In this regard, Koo *et al.* (30) showed that the ipsilateral kidney damage reduced slightly 10 days after RUUO in a 10-day UUO model. Therefore, finding the drugs that increase the kidney’s ability to renovate their functions after RUUO is a logical approach in clinical research (29). Accordingly, it seems that rapid RUUO can help to prevent further damage induced by the obstructed kidney. 

***RUUO model in animal***

RUUO model is considered as an appropriate model that provides an opportunity to explore the kidney inflammation associated with cellular-molecular processes related to tissue remodeling in the ipsilateral kidney (27). Also, RUUO model allows researchers to investigate the kidney recovery process and the ways to expedite this process (31). In this regard, some researchers eliminated the contralateral kidney to create a functional model (32, 33). Furthermore, this model is an inexpensive and reversible model and allows the researcher to evaluate the ipsilateral kidney function and histomorphology in animals (34). Also, the experimental RUUO model is an acceptable model for determining the treatment period and examining the effect of the novel treatment regimens (35). Overall, it seems that RUUO model allows clinicians to find a new intervention to prevent kidney injury.

***Endogenous renal repair after RUUO model***

It is known that the kidneys can process their endogenous repair from ligation at a molecular level in the RUUO mice (16). In this regard, it has become clear that RUUO after six weeks can decrease tubular injury, interstitial matrix expansion and also reduces the macrophages infiltration, renal fibrosis, and apoptosis in 10-day obstruction in mice (16). An experimental report has shown that post-RUUO relieving is dependent on the existence of non-atrophic nephrons in the renal medullary zone (36). In addition, after RUUO creation, tubular and glomerular plasticity cells and growth factors have been observed in the remodeling zone of the ipsilateral kidney (37). These factors are necessary for renal recovery progression (38) and consequently, the ipsilateral kidney can repair its GFR and urine concentration after RUUO (16). Moreover, the severity of obstruction is considered as an effective factor (39) in renal recovery after RUUO (Figure 1). However, it has been observed that GFR was not completely recovered 14 days of post-RUUO (17), as a result, it should not be expected that the GFR and RBF return to normal immediately after RUUO (31). In line with these studies, it has been shown that although renal interstitial volume and its tubular epithelium may increase immediately after RUUO (26), it gradually returns to normal (16). Some macrophages cytokines including interleukin-4 and interleukin-13 increase in post-RUUO kidney, and these mediators have beneficial responses such as intervention in cell survival, proliferation, angiogenesis (40, 41) and tubular epithelial cells regeneration (42). Furthermore, the histoarchitecture of the ipsilateral kidney will be recovered, and urinary output and its fractional excretion will return to normal conditions at six weeks post-RUUO (16). 

***The effect of post- RUUO period on ipsilateral kidney***

The renal structural and functional regenerative ability has been studied in the post-RUUO animal model (16). Since the duration of UUO plays an important role in renal regenerative ability; therefore, the urgent RUUO preserves the ipsilateral kidney from dysfunction (43). Also, a previous report showed that the renal functional and structural recovery is dependent on the time style of UUO in a pig model (35). In this regard, a study showed that GFR returns to normal after four weeks of RUUO in rats suffering from 3-day UUO (17). Moreover, it has been revealed that eighty-four percent of the renal glomerular and tubular cells show normal function after six weeks following RUUO (16). Furthermore, the renal interstitial collagen is increased about 2.4-fold in ipsilateral kidney suffering from 7-day UUO but normalized after 30 days post-RUUO (31). Also, interstitial expansion is decreased markedly after six weeks following RUUO in mice with 7-day UUO (31, 32). In addition, it is specified that obstructed kidney weight is reduced by 15%, 7 days after RUUO and returns to normal after 30 days of RUUO in 7-day UUO mice (35). It has also been reported that full recovery of the kidney will never be achieved if the RUUO does not occur promptly (16). However, the patient’s age and the status of contralateral kidney function also play an important role in obstructive kidney regeneration following RUUO (44). Also, animal model observations showed that the strain as another main indicator can interfere with renal recoverability during the RUUO period (33). Accordingly, it seems that if the RUUO is created faster, the chance of kidney recovery increases. However, the effect of post- RUUO period is also important (Figure 1).

## Discussion

It has been shown that relieving of the kidney function is completed at 14 days after RUUO in the animal with 3-day UUO, while kidney injury is continued even 28 days post-RUUO (17, 24). Moreover, it has been reported that after one year of RUUO, interstitial volume, macrophage infiltration, and fibrosis were markedly increased in the ipsilateral kidney (21). Therefore, RUUO may help to maintain the kidney function in the short term, but the fibrotic mechanisms caused by UUO ultimately lead to renal failure (24). Accordingly, since clinical interventions are performed after the diagnosis of obstruction (24); therefore, studies should be conducted to determine which medication or therapeutic approaches can help the kidney to fully recover. In the next topics, the therapeutic methods in the RUUO model are reviewed (Figure 1). 

***Mesenchymal stem cell therapy***

Bai *et al.* reported that arterially transplanted mesenchymal stem cells (MSCs) have a renoprotective effect and can degrade the renal interstitial fibrosis after RUUO model (Table 1) (45). Also, the fluorescence-based technique has shown that progressive renal tubular atrophy continues even post-RUUO, and administration of MSCs can attenuate the ipsilateral kidney injury and induce renal protection via inhibition of cell apoptosis in RUUO rats (Table 1) (45). Unlike this study, Semedo *et al.* showed that MSCs improve the histopathological status of the ipsilateral kidney in the RUUO model (46). Therefore, MSCs may be effective in reducing renal injury after RUUO creation.

***Non-steroid anti-inflammation drugs***

There are conflicting reports about the effects of non-steroid anti-inflammation drugs (NSAIDs) in the UUO and RUUO model (47, 48). In this regard, Hammad and his colleagues reported that the NSAIDs administration has a useful effect on GFR and RBF of the ipsilateral kidney at 2 weeks after RUUO in rats suffering from 5-day obstruction (48). Moreover, it has been observed that some NSAIDs reduced the renal interstitial fibrosis and tubular injury induced by UUO (48). Contrary to this, NSAIDs such as diclofenac sodium that is used to relieve the ureteral pain can decrease RBF via inhibition of prostaglandin synthesis (47, 49). In addition, the renal toxicity effect of NSAIDs was reported in other renal diseases (50, 51). Altogether, it seems that the use of NSAIDs is not logical in the RUUO model. However, this issue cannot be expressed with certainty (Table 1).

***L-Arginine***

It has reported that L-arginine can decrease the macrophage infiltration and has a renoprotective effect during the recovery phase of RUUO in 3-days UUO rats (Table 1) (24). Furthermore, N(ω)-nitro-L-arginine methyl ester (L-NAME) as a nitric oxide synthase (NOS) inhibitor decreases the renal function and increases the renal injury in rats with RUUO model. Indeed, these results suggest that nitric oxide (NO) has a beneficial effect on renal function after RUUO (Table 1) (24). In addition, accelerating in the NO-dependent signaling (via cyclic guanosine monophosphate pathway) significantly increases the renal recovery after RUUO and decreases kidney injury in a gender difference manner (52). Nevertheless, L-arginine supplementation acts as a pro-inflammatory or pro-fibrotic agent when administered coincident with increasing activity of inducible NOS (53). Accordingly, the L-arginine administration attenuates the renal function in rats with RUUO model, if inducible NOS is not stimulated at the same time. 

***Bone morphogenetic protein-7***

Bone morphogenetic protein-7 (BMP-7) as an anti-fibrotic agent was used in the RUUO surgical procedure to imitate the clinical condition (Table 1) (17). It has been revealed that transforming growth factor (TGF) has an important role in the pathogenesis of kidney damage (54), and BMP-7 inhibits the biological actions of TGF (55). In this regard, Morrissey *et al.* reported that BMP-7 improves GFR, interstitial volume, and fibrosis of the kidney with an RUUO condition (26). Totally, it seems that simultaneous treatment with an antifibrotic agent such as BMP-7 can help the renal recovery process in the RUUO model.

***Epidermal growth factor***

Epidermal growth factor inhibits the renal interstitial apoptosis and fibrosis in RUUO model and has renoprotective effect after relief of obstruction (Table 1) (56).

***Allopurinol***

It has been determined that the allopurinol administration before the relief of obstruction can increase the antioxidant system reinforcement in the RUUO animal model (Table 1) (57). Also, allopurinol increases the malondialdehyde (MDA) and glutathione (GSH) levels in ipsilateral kidney and scavenges free radical in this model (25). Since the animal studies reported a renoprotective effect for allopurinol in the RUUO model, this issue can be used in clinical practice.

***Losartan***

It has been reported that losartan as an angiotensin receptor subtype 1 blocker promotes the recoverability of kidney function after RUUO in a dog model (29). In this regard, our previous study showed that losartan administration (5 mg/kg body weight) increases the RBF and decreases the renal vascular resistance (RVR) in the ipsilateral kidney in RUUO animal model (Table 1) (15). Also, the renoprotective effects of losartan have been observed in the UUO model, so that administration of losartan decreases the RVR, and increases the RBF and GFR (Table 1) (58). In addition, losartan can decrease oxidative stress (59), macrophage infiltration (60), and renal injury in the UUO model (12). Besides, it has been reported that losartan attenuates the renal fibrosis and interstitial volume in the UUO model (58, 61). Totally, it seems that losartan may be effective in accelerating the ipsilateral renal recovery after RUUO. 

***PD123319***

PD123319 as an angiotensin receptor subtype 2 blocker increases the renal injury in the RUUO rat model (Table 1). This indicated that the angiotensin receptor subtype 2 plays a protective role in the obstructive kidney (15). Since the expression of this receptor is low in the kidneys (15); therefore, the stimulatory or inhibitory angiotensin receptor subtype 2 does not appear to be an effective therapeutic agent in the RUUO model.

***A779***

Angiotensin (1-7) via the Mas receptor has a renoprotective effect in the ipsilateral kidney of the RUUO model (Table 1) (62). A779 as an angiotensin (1-7) receptor blocker can reduce the angiotensin (1-7) biological effects (12, 62). Totally, the Mas receptor may be a milestone for clinical studies related to obstructive kidney recovery.

***Enalapril***

Enalapril is an angiotensin-converting-enzyme inhibitor (63) that decreases renal dysfunction and increases the renal recoverability after RUUO (Table 1) (64). Moreover, it has been demonstrated that enalapril has a renoprotective effect and attenuates renal injury in the UUO model (65).

***Aliskiren***

Aliskiren as a renin blocker has beneficial effects on the renal hemodynamic and functional parameters and attenuates the kidney injury in the RUUO model (Table 1) (66). The renin-angiotensin system (RAS) has many interactions with other factors in the kidney (12). Collectively, these reports emphasize that Aliskiren and other RAS antagonists have a renoprotective effect in the RUUO model.

***Bosentan***

Bosentan as a non-selective endothelin A/B receptor blocker increases the RBF and GFR after relief of the ureteral obstruction (Table 1) (67). Furthermore, it has been reported that the Bosentan decreases the actions of angiotensin II via the down-regulation of angiotensin subtype 1-receptors in vascular endothelium (12). Totally, Bosentan can reduce the endothelin-negative effects during UUO and after RUUO.

***The renal recoverability in RUUO patient***

Clinical reports revealed that after RUUO, approximately four weeks are needed to recover the kidney function in patients with ureteral obstruction (71). Moreover, the urinary proton (H^+^) excretion is incomplete in a patient with RUUO and systemic acidosis may develop (31). In addition, age as a factor can change the renal recovery rate in RUUO patients, so that the renal recovery rate is higher in younger patients (72). In clinical studies, there are a number of strategies that referred the approaches listed as below.

***Surgery***

It has been reported that surgery as an effective strategy can be useful in relieving chronic ureteral obstruction (73). In addition, ultrasonography may help to correct decision of the surgery (73, 74). Sometimes it may even require a nephrectomy; therefore, it may be useful to perform some tests for final confirmation (73). It seems that surgery may be useful in patients with chronic partial obstruction (Table 1).

***Stent***

It has been revealed that ureteral tumors can obstruct the urinary tract, and stents were applied in patients with malignant ureteral obstruction (75). Because this method is very invasive, patients rarely agree to do it (75). Moreover, urethrotomy, balloon dilatation, and stenting may be effective options for the treatment of stricture diseases (Table 1) (68, 69). In addition, urethroplasty is one of the main options with more success rate and higher satisfaction (Table 1) (70).

UUO: Unilateral ureteral obstruction, RUUO: Unilateral ureteral obstruction removal

**Table 1:** The main therapeutic factors associated with unilateral ureteral obstruction removal in animal models and human patients

Medication therapy (Method/molecule)	Targeting technique	Effects	References
Animal:			
**- MSCs ** **- NSAIDs** **- L-Arginine** **- BMP-7** **- EGF** **- Allopurinol** **- Losartan** **- PD123319** **- A779** **- Enalapril** **- Aliskiren** **- Bosentan**	Administration Administration Administration Administration Administration Administration AT_1_R blocker AT_2_R blocker Mas blocker ACE inhibitor Renin inhibitor Endothelin receptors antagonist	Decreased renal fibrosis, inhibited apoptosis Decreased inflammation, increased renal toxicity Decreased macrophage infiltration, improved renal function Decreased fibrosis, increased GFR Inhibited renal interstitial apoptosis and fibrosis Reinforced renal antioxidant system Increased RBF and decreased RVR, oxidative stress and macrophage infiltration parameters Increased RVR and decreased RBF Reduced angiotensin 1-7 biological effects, induced kidney injury Decreased renal dysfunction and increased renal recoverability Attenuated kidney injury Increased RBF and GFR	(45) (48) (24) (17) (56) (57) (58) (15) (62) (64) (66) (67)
Human: **- Urethrotomy** **- Stent and balloon dilatation** **- Urethroplasty**	Operation Operation Operation	Decreased renal interstitial pressure Applied in malignant ureteral obstruction Applied in malignant or chronic ureteral obstruction	(68) (69) (70)

MSCs: mesenchymal stem cells, NSAIDs: Non-steroid anti-inflammation drugs, BMP-7: Bone morphogenetic protein-7, EGF: Epidermal growth factor, AT_1_R: Angiotensin subtype 1 receptor, AT_2_R: Angiotensin subtype 2 receptor, ACE: Angiotensin converting enzyme, RBF: Renal blood flow, RVR: Renal vascular resistance, RUUO: Unilateral ureteral obstruction removal

## Conclusion

RUUO is the most important factor in preventing further damage to the obstructed kidney. However, the renal injury may occur after RUUO. Moreover, the patient’s age, in addition to pre- and post- RUUO period are important factors in ipsilateral kidney regeneration following RUUO. Therefore, medication therapy seems to be necessary after RUUO. In aggregation, the effect of some pharmacological agents was surveyed in the animal RUUO model. So that the MSCs (reducing renal injury), L-arginine (attenuates the renal function), BMP-7 (inhibits the renal fibrosis), epidermal growth factor (inhibits the renal apoptosis and fibrosis), Losartan (accelerating the renal recovery), angiotensin receptor subtype 2, angiotensin (1-7) and Mas receptor (plays a renoprotective role), Enalapril, Aliskiren and other RAS antagonists (attenuates the renal injury) and Bosentan (reduce the endothelin-negative effects) have renoprotective effect in RUUO animal model. In humans, surgery is the main strategy for RUUO creation via urethrotomy, urethroplasty, stent balloon dilatation, and stenting. 
